# A Systematic Review to Manage Avoidant/Restrictive Food Intake Disorders in Pediatric Gastroenterological Practice

**DOI:** 10.3390/healthcare11162245

**Published:** 2023-08-10

**Authors:** Ugo Cucinotta, Claudio Romano, Valeria Dipasquale

**Affiliations:** Pediatric Gastroenterology and Cystic Fibrosis Unit, Department of Human Pathology in Adulthood and Childhood “G. Barresi”, University of Messina, 98124 Messina, Italy; ugocucinotta@gmail.com (U.C.); romanoc@unime.it (C.R.)

**Keywords:** avoidant/restrictive food intake disorder, ARFID, children, pediatric gastroenterology, nutritional rehabilitation

## Abstract

Avoidant/Restrictive food intake disorder (ARFID) is a feeding disorder characterized by persistent difficulty eating, such as limited choices of preferred foods, avoidance or restriction of certain foods or food groups, and negative emotions related to eating or meals. Although ARFID mainly affects children, it can also occur in adolescents and adults. ARFID can have serious physical and mental health consequences, including stunted growth, nutritional deficiencies, anxiety, and other psychiatric comorbidities. Despite its increasing importance, ARFID is relatively underrecognized and undertreated in clinical practice. Treatment consists of a multidisciplinary approach involving pediatric gastroenterologists, nutritionists, neuropsychiatrists, and psychologists. However, there are several gaps in the therapeutic approach for this condition, mainly due to the lack of interventional trials and the methodological variability of existing studies. Few studies have explored the nutritional management of ARFID, and no standardized guidelines exist to date. We performed a systematic literature review to describe the different nutritional interventions for children and adolescents diagnosed with ARFID and to assess their efficacy and tolerability. We identified seven retrospective cohort studies where patients with various eating and feeding disorders, including ARFID, underwent nutritional rehabilitation in hospital settings. In all studies, similar outcomes emerged in terms of efficacy and tolerability. According to our findings, the oral route should be the preferred way to start the refeeding protocol, and the enteral route should be generally considered a last resort for non-compliant patients or in cases of clinical instability. The initial caloric intake may be adapted to the initial nutritional status, but more aggressive refeeding regimens appear to be well tolerated and not associated with an increased risk of clinical refeeding syndrome (RS). In severely malnourished patients, however, phosphorus or magnesium supplementation may be considered to prevent the risk of electrolyte imbalance, or RS.

## 1. Introduction

Avoidant/Restrictive food intake disorder (ARFID) is a feeding disorder characterized by persistent and severe difficulty eating or an apparent lack of interest in eating [1]. It primarily affects children but can also occur in adolescents and adults [2].

In recent years, there has been increasing awareness and recognition of ARFID as a clinical entity [2]. In 2013, it was included in the 5th edition of the Diagnostic and Statistical Manual of Mental Disorders (DSM-5) as a formal diagnostic category, and more recently in the 11th Revision of the World Health Organization’s International Classification for Diseases (ICD-11) [1,3].

According to the DSM-5, suspicion of ARFID arises when there is a persistent failure to meet nutritional and/or energy needs (Criterion A) that results in one (or more) of the following consequences [1]: (i) significant weight loss (or failure to achieve expected weight gain or inadequate growth in children); (ii) significant nutritional deficit; (iii) functioning dependent on enteral nutrition (EN) or oral supplements; and (iv) marked interference with psychosocial functioning.

To be diagnosed with ARFID, a person presenting with characteristics of criterion A must meet at least one of the four diagnostic criteria and fulfill all the so-called exclusion criteria (B-C-D): the disorder must not be explained by the unavailability of food or a culturally sanctioned practice (Criterion B), it must not occur exclusively together with anorexia nervosa (AN) and bulimia nervosa (BN), and there must be no evidence that food avoidance is a consequence of fear of gaining weight and over-assessment of weight and body shape (Criterion C) [1]. Finally, the disorder must not be due to a medical comorbidity or be explained by another mental disorder (Criterion D).

Based on the clinical presentation, ARFID can be further categorized into 3 subtypes [1,4]: (i) food avoidance due to an apparent lack of interest in food (a condition also referred to as emotional food avoidance disorder); (ii) sensory avoidance of food due to its sensory characteristics such as appearance, smell, texture, taste, or temperature (known as sensory sensitivity); (iii) food avoidance due to fear of aversive consequences of eating, such as choking, vomiting, or nausea.

ARFID can result in a wide range of health problems, including malnutrition, nutritional deficiencies, poor linear growth, dependence on tube feeding or high-energy food supplements, hospitalization for nutritional rehabilitation, and a significant impact on the quality of life (QoL) of both children and their families [5,6,7,8]. Not infrequently, children and adolescents with ARFID may exhibit signs and symptoms commonly seen in primary care and gastroenterology, such as abdominal pain, vomiting, or low weight and failure to thrive, which may coexist with ARFID and mask this condition [9,10]. Treatment typically involves a multidisciplinary approach that addresses both the physical and psychological aspects of the disorder [10,11,12]. However, there are several gaps in the therapeutic approach for patients with ARFID, mainly due to the lack of interventional trials and the methodological variability of existing studies [12]. Unlike eating disorders (EDs) such as AN, there have been few studies exploring the nutritional approach for patients with ARFID, and no standardized guidelines exist to date. The aim of this study was to conduct a systematic review of the literature to describe the different nutritional interventions available for children and adolescents diagnosed with ARFID and evaluate their efficacy and tolerability.

## 2. Methods

### 2.1. Information Sources and Search Strategy

This systematic review of the literature was conducted according to the preferred reporting items for systematic reviews and meta-analyses (PRISMA) guidelines [13]. The literature search was performed in May 2023 through MEDLINE via PubMed, SCOPUS, and Cochrane Library databases using the following string: (children OR infants OR adolescents OR pediatric) AND (ARFID OR Avoidant Restrictive Food Intake Disorder) AND (gastroenterology OR clinical presentation OR enteral nutrition OR nutrition OR nutritional rehabilitation OR nutritional management OR nutritional approach OR oral supplements OR diet OR dietary management).

The searches were conducted to answer the research question directly, which was developed in the population, intervention, and outcome (PIO) format. The clinical question was as follows: “what are the efficacy and tolerability (O) of different nutritional interventions (I) in children and adolescents diagnosed with ARFID (P)?”.

### 2.2. Study Selection

Articles published between January 2013 and May 2023 were identified. ARFID was first included as a diagnosis in the DSM-5, which was published in 2013. Therefore, 2013 was chosen as the earliest date for the search.

The inclusion criteria were: articles written in English, belonging to the categories of clinical study, clinical trial, clinical trial protocol, multicenter study, randomized controlled trial, and observational study, which reported the therapeutic approach to ARFID in a pediatric population (age < 18 years) with a clear description of nutritional/dietary strategies or interventions, either meal-based, enteral nutrition via nasogastric (NG), nasojejunal (NJ), or gastrostomy tube, parenteral nutrition (PN), or oral supplements.

Articles were excluded by title, abstract, or full text if (i) they belonged to the categories of review, systematic review, meta-analysis, case reports, and case series; (ii) they included only a non-pediatric population (age ≥ 18 years); and/or (iii) they were irrelevant to the investigated issue. Articles including topics such as anorexia nervosa, bulimia nervosa, neophobia, non-better specified EDs or selective feeding, functional dysphagia, and articles that discuss changes in DSM-5 diagnostic criteria were then excluded. Studies that only focused on behavioral or pharmacological interventions were also excluded.

The titles and abstracts of all retrieved articles were independently screened for relevance by two authors. The suitability of all full-text articles was then assessed by all authors.

### 2.3. Analyses and Endpoints

A quality assessment of the studies was conducted in order to assess their most important biases and weaknesses. The Newcastle–Ottawa scale was used to assess the quality of the observational studies [14]. This scale evaluates the studies according to the comparability of the results, the selection of the population and the controls, and the reliability of the outcomes. Eventual discrepancies in the quality assessment were discussed and resolved by two independent authors. We rated the quality of the studies (good, fair, and poor) by awarding stars in each domain following the guidelines of the Newcastle–Ottawa Scale. A “good” quality score required 3 or 4 stars in selection, 1 or 2 stars in comparability, and 2 or 3 stars in outcomes. A “fair” quality score required 2 stars in selection, 1 or 2 stars in comparability, and 2 or 3 stars in outcomes. A “poor” quality score reflected 0 or 1 star(s) in selection, 0 stars in comparability, or 0 or 1 star(s) in outcomes. Only studies with a good-quality evaluation were included in this review.

From each selected study, the following data were extracted using a predefined database: authors’ names, year of publication, study design, characteristics of included patients (i.e., age, gender, signs and symptoms at hospital admission), route of administration of the nutritional intervention (oral nutrition, EN via NG/NJ tube or gastrostomy tube, PN), caloric intake at the start of the nutritional intervention and rate of caloric increase, anthropometric parameters at the start and at the end of the nutritional intervention, including weight, percentage ideal body weight, and body mass index, standardized mean differences, and rate and type of adverse events associated with feeding resumption.

Key outcomes of interest included the type and characteristics of the nutritional interventions used, their effectiveness in terms of weight restoration, and the occurrence of adverse events.

## 3. Results

**Figure 1:**
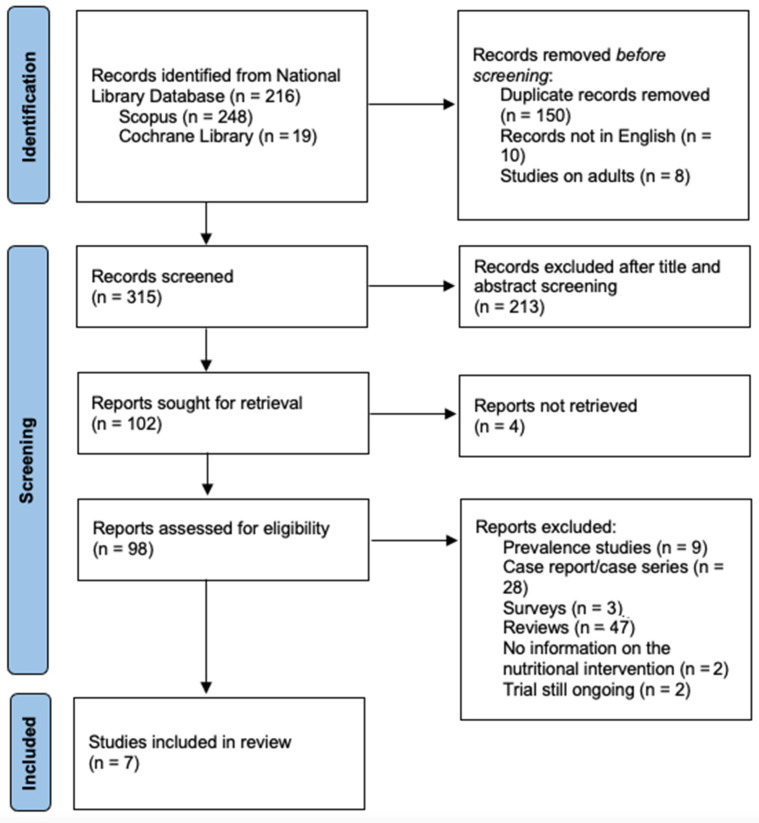
PRISMA flow diagram for systematic reviews [13].

A total of 483 publications were initially retrieved (Figure 1). One hundred fifty were duplicates and were excluded. Another 10 studies were excluded because they were not in English and eight because they were conducted on adults. Of the remaining 315 studies, 213 were excluded based on title and abstract screening because they were not relevant to the outcome considered. Of the reports retrieved, 91 were further excluded because they were either reviews, prevalence studies, case reports or series with fewer than 10 patients (<10), studies without specified nutritional interventions, or studies that were still ongoing. Finally, seven retrospective studies remained to be included in the current analysis [15,16,17,18,19,20,21]. All included studies were published between 2013 and 2021 and described a total of 147 patients with ARFID who received nutrition interventions in an inpatient setting (Table 1). No studies were identified that described specific nutrition interventions in the outpatient setting.

Primary and secondary outcomes varied slightly across studies but were mainly focused on the efficacy and tolerability of various nutritional interventions applied to children and adolescents with moderate-to-severe malnutrition and diagnosed with EDs (including ARFID) in inpatient settings.

### 3.1. Clinical Characteristics of the Patients

The ARFID patients in the studies were predominantly female (70–100%), with a mean age ranging from 10.7 to 19.1 years.

At admission, the symptoms most frequently reported were abdominal pain, nausea, vomiting, or fear of vomiting [16,18,19,21], while the most frequent clinical signs were bradycardia [18,19,21] and orthostatic instability [21]. In three studies, the aversive subtype was the predominant presentation of ARFID (78% to 92% of patients) [15,16,17].

All patients presented with moderate-to-severe malnutrition at admission. In studies using percentage ideal body weight (%IBW) as the main parameter of nutritional status, mean %IBW ranged from <75% to 78.7% [15,18,19,20], while in those using body mass index (BMI) z-score, mean BMI z-score ranged from −2.2 to −1.6 [16,17,21].

### 3.2. Characteristics of Nutritional Interventions

Except for two studies where data on caloric intake were not reported [15,21], the range of the initial caloric intake was relatively wide and set between 1200 kcal and 2500 kcal per day [16,17,18,19,20] (Table 1). The decision to start with lower caloric regimens (typically 1200 kcal/day) was mainly driven by a lower %IBW at admission or if extreme dietary restriction was reported (e.g., <500 kcal/day for several weeks) [19,20]. Daily caloric intake was then titrated, increasing by 200–400 kcal/day to achieve 0.2–0.3 kg of weight gain per day and/or an overall goal of 1–2 kg of weight gain per week [18,19,20].

Only one study compared the efficacy of lower (<1500 kcal/day) versus higher (>1500 kcal/day) calorie regimens in a heterogeneous group of adolescents with EDs, showing no significant differences in %IBW increase but a shorter length of stay (LOS) in those treated with higher calorie regimens [20].

In all but one study [15,16,18,19,20], the initial route of administration described for the nutritional intervention was orally. In most cases, the initial meal plan was prescribed by a dietitian based on diet history and clinical presentation. It generally consisted of three daily meals and rotating menus to increase food choice and willingness to eat, with or without the introduction of snacks during the day. Unconsumed calories were replaced with a high-calorie supplement (either at 1.0 kcal/mL or 1.5 kcal/mL concentration) or, if orally refused, via a nasogastric tube through intermittent or continuous enteral feeding [15,16,18,19,20].

Few data comparing the efficacy of oral versus enteral nutrition are available. In one study [18], those who received primarily EN were started on lower calories than patients who received primarily oral nutrition (1500 kcal vs. 2200 kcal/day), resulting in slower weight gain (0.2 kg/day vs. 0.4 kg/day) and longer hospitalizations. In another study [20], higher-calorie diets administered via NG/NJ tubes were not associated with shorter LOS. However, both studies observed that patients needing NG/NJ tubes typically had significant behavioral components to their food refusal that required more prolonged behavioral intervention. They also had medical complications (e.g., superior mesenteric artery syndrome, vomiting) that postponed the start of safe oral feeding, thus increasing the LOS.

Only one study was focused on the inpatient management and clinical outcomes of children with EDs who primarily received total parenteral nutrition (TPN) [17]. The indications for TPN treatment included cases of severe malnutrition, dehydration, electrolyte disorders, hypoglycemia, and heart failure. The TPN energy intake on hospital admission started at 2090 kcal per day for the first week and was increased every week to ensure acceptable weight gain with a combination of both oral feeding and PN. TPN was overall well tolerated, and the average BMI z-score increased from −2.2 to −1.1 at discharge.

### 3.3. Refeeding-Related Adverse Events

All patients underwent daily monitoring of body weight and received continuous cardiac monitoring, at least for the first few days of recovery. Only three studies reported the occurrence of refeeding-related adverse events [18,19,20]. In one of them [18], all patients received phosphorus supplementation twice daily for 5 days to prevent the occurrence of refeeding syndrome (RS), while in the other two, no electrolyte supplementation was routinely administered unless decreases in serum electrolytes were identified [19,20]. In the three studies, a variable rate between 14% and 57% of patients had documented electrolyte shifts during the first 72 h after admission, including hypophosphatemia, hypomagnesemia, and hypokalemia, but no cases of clinical RS occurred in any of them [18,19,20]. One of these studies compared the tolerability of higher (>1500 kcal/day) versus lower (<1500 kcal/day) calorie regimens in terms of the occurrence of adverse events and showed that the risk of initial hypophosphatemia was not associated with the initial calorie level or rate of caloric advancement but rather with the initial %IBW [20].

### 3.4. Co-Interventions and Criteria for Discharge

Alongside the refeeding protocol, all patients in the included studies variably received a multidisciplinary approach based on psychological, psychiatric, and behavioral interventions, and all physicians involved worked together to reinforce psychoeducation around the feeding disorders and their management.

Criteria for discharge varied slightly across the reports; in two studies, they were mainly represented by weight-goal achievement, resolution of bradycardia, hypotension, and hypothermia, and the absence of electrolyte abnormalities. Patients on nasogastric tube feeds were generally transitioned to oral nutrition before discharge and then addressed in outpatient treatment sessions [18,19]. In another study, patients were considered in remission and subsequently discharged when their behavioral eating patterns were restored and they could maintain their target weight for ≥2 weeks [15]. In the other studies, the main criteria for discharge generally consisted either of clinical improvement or weight restoration. In most cases, the follow-up program consisted of weekly outpatient treatment sessions administered by trained psychiatrists to maintain the target body weight and normalize eating patterns.

## 4. Discussion

This systematic review discusses the latest evidence from the literature on the efficacy and tolerability of different nutritional protocols in children and adolescents with ARFID who are hospitalized for nutritional rehabilitation. Several gaps still exist in the therapeutic approach for patients with ARFID, mainly related to the methodological incompleteness of the studies conducted to date and the lack of interventional studies. Our systematic review is in line with a recent scoping review [12] showing that studies conducted from 2009 to 2019 on EDs are mostly non-experimental and descriptive, with no reports of interventions or long-term (>6 months) follow-up. Another gap in the current treatment of ARFID is the over-compartmentalization of approaches to Eds, which historically have been treated from discipline-specific perspectives (e.g., gastroenterologists, nutritionists, speech/language pathologists, occupational therapists, and psychologists) [12].

The studies included in our review were conducted in hospital settings where patients received a multidisciplinary team (MDT) approach consisting of various nutritional interventions for acute management of malnutrition, along with different psychological, behavioral, and pharmacological supports.

The indication for hospital admission was mostly represented by moderate-to-severe malnutrition, with or without medical instability, and a lack of oral intake. This is in line with the position paper from the Society for Adolescent Health and Medicine (SAHM) for the medical management of restrictive EDs, which proposes to consider hospitalization whenever one or more of these conditions occur [22]:

1. ≤75% of IBW for age and sex;
2. Dehydration or electrolyte disturbance (hypokalemia, hyponatremia, hypophosphatemia);
3. Electrocardiogram abnormalities (e.g., prolonged QTc or severe bradycardia);
4. Severe bradycardia (heart rate < 50 beats/min daytime); hypotension (<90/45 mm Hg); hypothermia (body temperature < 35.6 °C); orthostatic increase in pulse (>20 beats/min) or decrease in blood pressure (>20 mm Hg systolic or >10 mm Hg diastolic);
5. Arrested growth and development;
6. Failure of outpatient treatment;
7. Acute food refusal;
8. Uncontrollable bingeing and purging;
9. Acute medical complications of malnutrition (e.g., syncope, seizures, cardiac failure, pancreatitis, and so forth);
10. Comorbid psychiatric or medical condition that prohibits or limits appropriate outpatient treatment (e.g., severe depression, suicidal ideation, obsessive-compulsive disorder, type 1 diabetes mellitus).

For weight restoration in patients with moderate to severe malnutrition secondary to restrictive EDs, it has been historically recommended to start with low-calorie diets (<1500 kcals/day) to prevent refeeding syndrome (RS) [22,23,24].

RS is a potential severe complication that may occur during refeeding regimes, usually between 2 and 5 days after the reintroduction of calories [16]. The sudden availability of glucose leads to a rapid shift from a chronically catabolic state to an anabolic state, with inhibition of gluconeogenesis and an insulin surge. This causes the rapid consumption of low body stores of phosphorus, magnesium, and potassium and an intracellular shifting of these electrolytes, resulting in low serum electrolyte levels [20,25,26]. Clinical consequences may be severe, including muscle weakness and cramping, cardiac arrhythmias, vomiting, seizures, delirium, and death [25].

In recent years, several studies have been conducted in patients aged 10 to 21 years, mainly diagnosed with AN, to evaluate the efficacy and tolerability of diets with higher caloric content, especially in the case of moderate to severe malnutrition. Interestingly, they reported shorter hospital stays [27,28], faster weight gain [28,29,30], a low rate of hypophosphatemia during nutritional rehabilitation, and no incidence of clinical RS [27,28,29,30].

Despite the scarce number of studies investigating these outcomes in patients with ARFID, the present review is in line with these findings, suggesting that more aggressive refeeding regimens may lead to shorter LOS, are well tolerated, and are not associated with an increased risk of clinical RS [18,19,20]. Electrolyte shifts, especially in serum phosphate, potassium, and magnesium, can, however, be observed after the start of refeeding protocols, which may precede the development of a true RS if not corrected. In the studies reporting the occurrence of refeeding-related complications [18,19,20], patients did not receive routine prophylaxis against RS except for one [18], and electrolyte supplementation was only initiated when decreases in serum electrolytes were identified. In all cases, there was no standard protocol defining the timing of electrolyte supplementation. The attending physician used clinical judgment and often started electrolyte supplementation prior to serum levels falling into the abnormal range if levels were noted to be dropping rapidly. Phosphorus or magnesium supplementation may therefore be considered in severely malnourished patients to prevent the risk of electrolyte imbalance, or RS.

In terms of route of administration for the nutritional intervention, the present review suggests that the oral route can represent the first way of administration for the refeeding protocol and should always be preferred in the case of compliant patients and in the absence of contraindications [16,18,19,20,21].

Limited research exists on the use of enteral feeding via NG tube in patients with ARFID. Although an NG tube is commonly used in other medical conditions to support nutrition and growth, including severe AN [31,32], it should be generally considered a last resort (e.g., in cases of clinical instability) and is not recommended as a long-term solution [32]. It may be effective in improving weight gain and nutritional status in some ARFID patients, but it is important to consider the potential risks and benefits of this intervention on an individual basis. It can be distressing and uncomfortable for patients, and this is especially true for patients with high visceral and oral palatal sensitivity or those who may experience negative emotional and psychological consequences [32]. Therefore, the use of these interventions should be carefully considered as part of a comprehensive treatment plan that includes behavioral and nutritional interventions and support for the patient’s emotional and psychological well-being.

Extensive data also exists on the use of gastrostomy tube feeding for feeding and EDs [31,32]. Among the studies included in this review, only one reported the use of gastrostomy placement as a nutritional intervention, but no data on the number and characteristics of patients were available [21]. In a case series, three patients with food refusal secondary to a psychiatric illness unrelated to body image issues underwent gastrostomy tube placement with healthy weight restoration [32]. While these studies suggest that gastrostomy tube feeding may be an effective treatment option for severe cases of ARFID, it is important to note that this is a highly individualized decision that should be made in consultation with a healthcare provider. Gastrostomy tube feeding is a medical intervention associated with potential complications and should only be considered when all other treatment options have been exhausted and the benefits outweigh the risks.

In any case, medical hospital stabilization should be considered a short-term intervention to address acute medical needs by closely monitoring the patient and providing a structured therapeutic plan designed to address their specific nutritional needs [33,34]. Once the child’s physical health has been stabilized, the focus of treatment may shift to addressing the underlying psychological factors contributing to their ARFID [33,34,35]. This may involve transitioning the child to outpatient treatment, such as behavioral therapies [34,35,36].

Oral nutritional supplements (ONS) are not routinely recommended but can be used in combination with behavioral interventions, such as the food chaining technique, or to support the inpatient refeeding protocols to increase the caloric intake, compensate for any selective macro- and micronutrient deficits, support the patient’s overall progress, and reduce food-related anxiety [18,19,20,37].

## 5. Future Directions and Conclusions

While the role of nutrition in the management of ARFID is increasingly recognized, there is still a need to further explore and refine the understanding of this condition and to develop effective nutritional approaches and dietary interventions for individuals with ARFID. Limited evidence-based guidelines and protocols are currently available to guide physicians in this area.

A crucial aspect that needs further investigation is the effectiveness of different dietary interventions in the treatment of ARFID. Although the study included in this study reported the effectiveness of certain dietary interventions or dietary changes, these were purely retrospective, so the evidence base was relatively limited. There is a need for more rigorous clinical trials to evaluate the effectiveness of specific nutritional approaches and validate well-established nutritional rehabilitation programs. These studies should not only assess short-term outcomes but also long-term effects on nutritional status, weight recovery, eating behavior, and psychological well-being. By conducting such studies, we can gather robust evidence on the effectiveness of different nutritional interventions and establish best practices for their implementation in ARFID treatment.

Additionally, considering the sensory sensitivities, aversions, and food preferences commonly associated with ARFID, it is crucial to explore innovative strategies for addressing these challenges within the context of nutritional interventions. Incorporating sensory-based therapies, such as food exposure with sensory integration, can help individuals gradually become more comfortable with new textures, flavors, and food presentations. Furthermore, integrating behavioral and cognitive interventions into dietary counseling sessions can assist individuals in challenging their food-related fears, addressing cognitive distortions, and developing more positive attitudes towards food and eating. Exploring the effectiveness and feasibility of these integrated approaches can greatly enhance the success of nutritional interventions in ARFID treatment.

In summary, future research should focus on conducting robust clinical trials to evaluate the effectiveness of various nutritional interventions, including gradual exposure and nutritional rehabilitation programs. Longitudinal studies to assess the nutritional status of individuals with ARFID and the impact of nutritional deficiencies on symptoms are also needed. In addition, exploring innovative strategies that incorporate sensory-based and behavioral/cognitive interventions into dietary counseling can provide comprehensive support for people with ARFID and help them develop healthier relationships with food and achieve improved nutritional well-being.

## Figures and Tables

**Table 1:** Studies describing nutritional interventions in children and adolescents diagnosed with ARFID [15,16,17,18,19,20,21].

References	Design and Patients	Clinical Characteristics	Nutritional Intervention	Adverse Events	Outcomes/Other Findings
Strandjord et al. (2015) [18] USA	Retrospective To compare patients with different EDs hospitalized for acute medical stabilization (n = 244) in terms of presentation, treatment response and 1-year outcomes. ARFID patients (n = 41), 85% female, mean age 16 years.	Mean %IBW on admission 78% Patients with ARFID had less weight loss, comorbidity, and bradycardia than AN patients at admission.	Initial caloric intake between 1500 and 2300 kcal/day orally. Increase of 200 kcal per day. Goal: 0.2 kg weight gain per day. Unconsumed calories: replaced with high-calorie supplement drinks. If refused orally, given via NG tube.	No patient experienced RS During refeeding n = 2 experienced hypokalemia n = 1 hypomagnesemia n = 1 hypophosphatemia	Mean increase in %IBW during hospitalization: 15% ARFID and AN patients had similar outcomes 1 year after initial admission ARFID patients required more EN and longer hospitalizations than AN
Maginot et al. (2017) [20] USA	Retrospective Patients with EDs hospitalized for medical rehabilitation (n = 87) ARFID patients (n = 10), 100% female, mean age 14.6 years	Mean %IBW on admission 78.7% 29% of the entire cohort was severely malnourished (<75% IBW)	Initial caloric intake between 1000 and 3000 kcal/day orally (3 meals ± 3 snacks). ~1200 kcal/day if patient severely malnourished. Then adjusted to goal: 0.15–0.3 kg weight gain/day. If refused orally, NG tube.	Up to 57% of patients experienced hypophosphatemia and up to 52% hypomagnesemia during the first 72 h after admission	Increase in %IBW during hospitalization between 5% and 6.7% A higher calorie regimen was not associated with increased risk of hypophosphatemia, hypomagnesemia or hypokalemia
Peebles et al. (2017) [19] USA	Retrospective Patients with EDs admitted for a first hospitalization (n = 215), 88% female, mean age 15.3 years. ARFID patients n = 9	84.2% of the entire cohort met criteria for severe malnutrition (<75% IBW)	Initial caloric intake between 900 and 2800 kcal/day via 7 days rotating menus. Increase of 200–400 kcal/day until goal calories. If repetitively refused orally, given via NG tube.	No patient experienced RS 14% received phosphorus supplementation for refeeding hypophosphatemia, 4% potassium supplementation and 3% magnesium supplementation.	Mean increase in %IBW during hospitalization: 5% Patients averaged 100.9 %IBW at 4-weeks follow-up. Just 3.8% were rehospitalised in the 30 days after discharge.
Makhzoumi et al. (2019) [16] USA	Retrospective Patients with EDs admitted to an integrated hospital-based treatment programme (n = 275, 86% female) ARFID patients (n = 27, mean age 19.1)	Mean BMI at admission: 16.5 (−2 z-score) More common GI symptoms at admission: abdominal pain, GERD, vomiting. 78% of ARFID presented with an aversive subtype	3 varied meals/day started on 1200–1500 kcal/day. Caloric increases every 2 days (target calories 3500–4000 kcal/day by day 10–12). Calories above 2500/day administered via nutritional supplements.	N.A.	Mean BMI at admission: 16.5 Mean BMI at discharge: 18.9 Mean inpatient weight gain rate: 1.36 kg/week
Kurotori et al. (2019) [15] Japan	Retrospective Patients with EDs hospitalized for nutritional rehabilitation (n = 92). ARFID patients (n = 13, 85% females, mean age 10.7 years)	Mean %IBW on admission 74% 8 (61.5%) were severely malnourished (<75% expected BW) 92% of ARFID presented with an aversive subtype	Meals administered orally (no data available on the caloric intake). Enteral nutrition via NG tube in case of persistent food refusal.	N.A.	Mean increase in %IBW during hospitalization: 4.9%
Tsang et al. (2020) [21] USA	Retrospective ARFID patients hospitalized for nutritional rehabilitation (n = 38, 68% females, mean age 12.8 years).	Average %IBW on admission 85.9. Mean BMI z-score on admission: −1.66. Most reported GI symptoms: abdominal pain, nausea, vomiting.	Almost half of patients (47.4%) required enteral feeds (i.e., via nasogastric, nasojejunal, or gastrostomy tube). No data on the caloric intake.	N.A.	Average %IBW on admission 85.9, Average %IBW on discharge 87.6.
Tamura et al. (2021) [17] Japan	Retrospective Elementary-school children hospitalized for refractory EDs started on TPN (n = 22). ARFID patients (n = 9), 78% females, mean age 11.5 years	Mean BMI z-score at admission −2.2 89% of ARFID presented an aversive subtype	All patients started on TPN with 2090 kcal/day for the first week, then increased every week (by increase of PN and introduction of oral feeding) to ensure acceptable weight gain. Enteral nutrition via NG tube if persistent oral intake refusal.	N.A.	Mean BMI z-score at admission −2.2; at discharge −1.1. No significant differences in weight gain between ARFID patients and AN patients

ARFID, avoidant/restrictive food intake disorder; AN, anorexia nervosa; BMI, body mass index; BW, body weight; GERD, gastroesophageal reflux disease; ED, eating disorder; EN, enteral nutrition; NA, not available; NG, nasogastric; %IBW, percentage ideal body weight; RS, refeeding syndrome; TPN, total parenteral nutrition.

## Data Availability

The dataset used during the current study is available from the corresponding author on reasonable request.
